# Defining the conformation of human mincle that interacts with mycobacterial trehalose dimycolate

**DOI:** 10.1093/glycob/cwu072

**Published:** 2014-07-15

**Authors:** Sabine A F Jégouzo, Edward C Harding, Oliver Acton, Maximus J Rex, Andrew J Fadden, Maureen E Taylor, Kurt Drickamer

**Affiliations:** Department of Life Sciences, Imperial College, Sir Ernst Chain Building, London SW7 2AZ, UK

**Keywords:** C-type lectin, glycan-binding receptor, glycolipid, mincle, *Mycobacterium tuberculosis*

## Abstract

Trehalose dimycolate, an unusual glycolipid in the outer membrane of *Mycobacterium tuberculosis*, stimulates macrophages by binding to the macrophage receptor mincle. This stimulation plays an important role both in infection by mycobacteria and in the use of derivatives of mycobacteria as adjuvants to enhance the immune response. The mechanism of trehalose dimycolate binding to the C-type carbohydrate-recognition domain in human mincle has been investigated using a series of synthetic analogs of trehalose dimycolate and site-directed mutagenesis of the human protein. The results support a mechanism of binding acylated trehalose derivatives to human mincle that is very similar to the mechanism of binding to bovine mincle, in which one glucose residue in the trehalose headgroup of the glycolipid is ligated to the principle Ca^2+^-binding site in the carbohydrate-recognition domain, with specificity for the disaccharide resulting from interactions with the second glucose residue. Acyl chains attached to the 6-OH groups of trehalose enhance affinity, with the affinity dependent on the length of the acyl chains and the presence of a hydrophobic groove adjacent to the sugar-binding sites. The results indicate that the available crystal structure of the carbohydrate-recognition domain of human mincle is unlikely to be in a fully active conformation. Instead, the ligand-binding conformation probably resembles closely the structure observed for bovine mincle in complex with trehalose. These studies provide a basis for targeting human mincle as a means of inhibiting interactions with mycobacteria and as an approach to harnessing the ability of mincle to stimulate the immune response.

## Introduction

Cells of the innate immune system, including macrophages and dendritic cells, express a range of glycan-binding receptors, many of which contain C-type carbohydrate-recognition domains (CRDs) (van Kooyk and Rabinovich 2008). A large subgroup of these receptors function in the capture of microorganisms, including viruses, bacteria, fungi and parasites, leading to internalization of these potential pathogens. Recognition is often based on binding of the sugars mannose and GlcNAc, which are more common on the pathogen surfaces than on host cells (Sukhithasria et al. 2013), but additional types of sugars are sometimes also targeted, including GalNAc on some species of bacteria (Jégouzo et al. 2013) and the Lewis^x^ epitope common on certain parasites (van Die and Cummings 2010). Uptake can be a direct form of innate immune protection, in which the microorganism is destroyed, and it can also inform the adaptive immune system by facilitating presentation of antigens to lymphocyte precursors (van Kooyk and Rabinovich 2008).

In addition to these well-established functions in pathogen clearance, there is increasing evidence that interaction of some ligands with glycan-binding receptors can initiate signaling within macrophages and dendritic cells. For example, binding of viruses and bacteria to the dendritic cell receptor DC-SIGN (dendritic cell-specific intercellular adhesion molecule-3 grabbing nonintegrin) and the macrophage galactose receptor have been shown to initiate kinase activation and cytokine release (Geijtenbeek and Gringhuis 2009; Napoletano et al. 2012; Svajger et al. 2010). However, details of how downstream signaling events are initiated from these receptors remain to be elucidated.

**Fig. 1.**
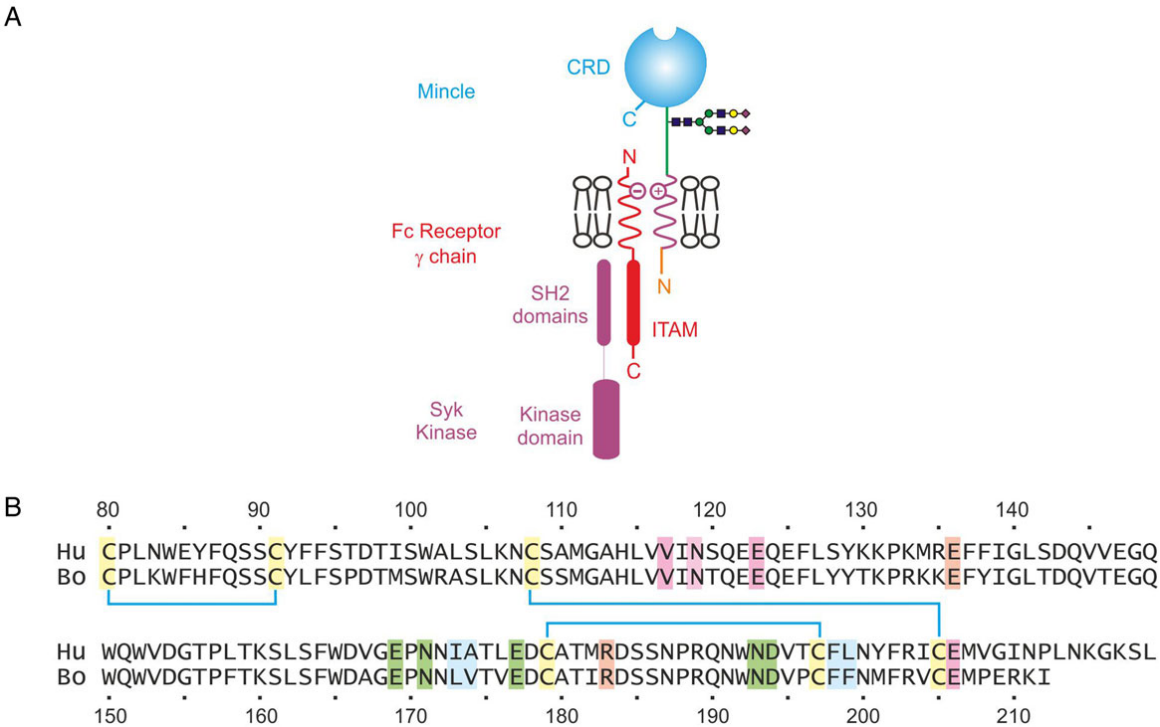
Organization of mincle. (**A**) Arrangement of mincle in the macrophage membrane, showing interactions with the common Fc receptor γ subunit within the lipid bilayer, which in turn interacts with Syk kinase on the cytoplasmic side of the membrane. ITAM, immunotyrosine activation motif. (**B**) Alignment of the amino acid sequences of the CRDs from human and bovine mincle. Disulfide bonds are indicated as cyan-colored lines between cysteine residues. Ligands for the principal Ca^2+^ are highlighted in green, ligands for the accessory Ca^2+^ are highlighted in violet, residues in the secondary sugar-binding site are highlighted in peach and residues that form the hydrophobic groove are highlighted in blue.

One of the best understood examples of a glycan-initiated signaling pathway results from stimulation of mincle, which is a simple type II transmembrane receptor expressed on macrophages (Matsumoto et al. 1999). The ligand-binding portion of mincle is a C-type carbohydrate-recognition domain that is attached to the cell surface by a short stalk and a transmembrane anchor. The receptor polypeptide forms a hetero-oligomer with the common γ-subunit of the Fc receptor, which interacts with Syk kinase through an immunotyrosine activation motif on the cytoplasmic side of the membrane (Figure 1A). Syk activation leads in turn to stimulation of the CARD9 signaling pathway (Yamasaki et al. 2008; Werninghaus et al. 2009).

The recent demonstration that trehalose dimycolate, an unusual glycolipid in the outer membrane of mycobacteria, is a ligand for mincle suggests that stimulation of the CARD9 pathway through mincle plays an important role in the interaction between mycobacteria and host macrophages (Ishikawa et al. 2009; Schoenen et al. 2010). In the case of bovine mincle, a mechanism for selective binding of the glucose-α1-1′α-glucose trehalose headgroup and fatty acid chains esterified to the 6-OH groups of the glucose residues has been suggested, based on crystal structures and binding of synthetic analogs of trehalose dimycolate (Feinberg et al. 2013). In the primary binding site, one glucose residue is ligated to the conserved Ca^2+^ characteristic of C-type CRDs, a second engages in a secondary binding site and acyl chains attached to the 6 OH group interact with a hydrophobic groove on the surface of the CRD. However, in spite of 77% sequence identity between the human and bovine CRDs (Figure 1B), structural analysis of the human form of mincle in the absence of bound ligand (Furukawa et al. 2013) suggests that it lacks some of the key features that would be involved in the interactions seen in the bovine CRD complex with trehalose. One of the amino acid side chains that ligates to the conserved Ca^2+^ is displaced away from the primary binding site and one side of the hydrophobic groove is not formed. These results raised the possibility that the human and bovine proteins might differ significantly in their mechanism of sugar binding.

In order to clarify the extent of similarity between the mechanisms of ligand interaction of the human and bovine forms of mincle, a series of binding and mutagenesis studies of the human protein have been conducted. The results demonstrate that, in keeping with the high degree of sequence identity between the two receptors, they interact with ligands in a very similar way.

## Results

### Binding of the CRD from human mincle to trehalose dimycolate

**Fig. 2.**
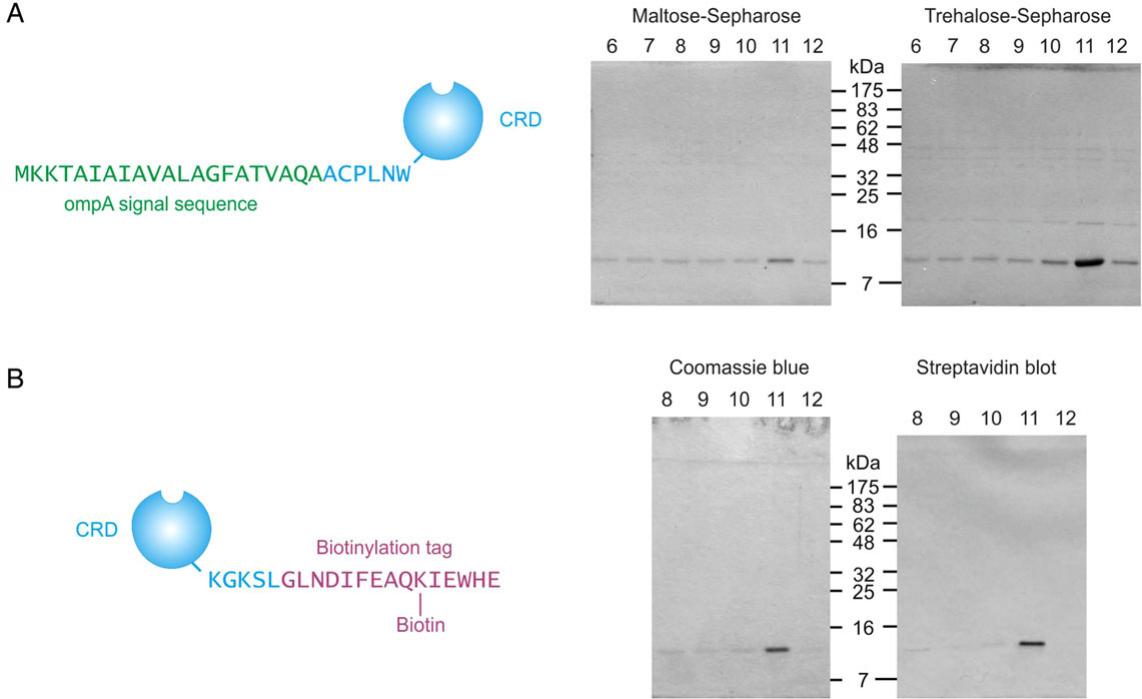
Expression and purification of human mincle CRD. (**A**) Expression of the mincle CRD with a bacterial signal sequence followed by direct purification of folded protein released from the cytoplasm. Purification was attempted on 10-mL columns of immobilized sugars that were washed with 14 mL of Ca^2+^-containing buffer and then eluted with 1 mL fractions of EDTA-containing buffer. Aliquots of the elution fractions were analyzed on SDS–polyacrylamide gels that were stained with Coomassie blue. (**B**) Purification of mincle CRD with a biotin tag following solubilization in guanidine and renaturation. Purification on trehalose-Sepharose was performed as in (A). Parallel gels were stained with Coomassie blue or blotted onto nitrocellulose and probed with alkaline phosphatase-conjugate avidin.

Bacterial expression systems were developed for analysis of the properties of the extracellular domain of human mincle. Initial experiments were undertaken using an expression system in which the CRD was secreted from bacteria under the direction of the ompA signal sequence. Following protocols used for other C-type lectins, induction of protein expression in the presence of high concentrations of Ca^2+^ was used to facilitate folding of the domain (Taylor and Drickamer 2003). Protein expressed in this way bound poorly to maltose-Sepharose columns, but most remained bound to trehalose-Sepharose columns during washing in the presence of Ca^2+^ and was eluted with EDTA (Figure 2A and B). However, in order to compensate for relatively weak binding, it was necessary to use an extended column of trehalose-Sepharose and to minimize washing, since some mincle CRD was eluted even in the presence of Ca^2+^. The results confirm that the CRD of human mincle binds to trehalose, but with modest affinity.

In order to facilitate binding studies, an alternative expression system was developed in which a biotinylation tag was added to the C-terminus of the CRD. Protein was expressed intracellularly, to ensure efficient biotinylation in the presence of cytosolic biotin ligase. The resulting inclusion bodies were solubilized in guanidine and renatured by dialysis against buffer containing Ca^2+^. The resulting protein could be purified on trehalose-Sepharose and showed the same characteristic leaching through the column as the protein produced directly without a tag (Figure 2C). The presence of the biotinylated tag was confirmed by blotting the protein onto nitrocellulose and probing the blots with alkaline phosphatase-conjugated avidin.

**Fig. 3.**
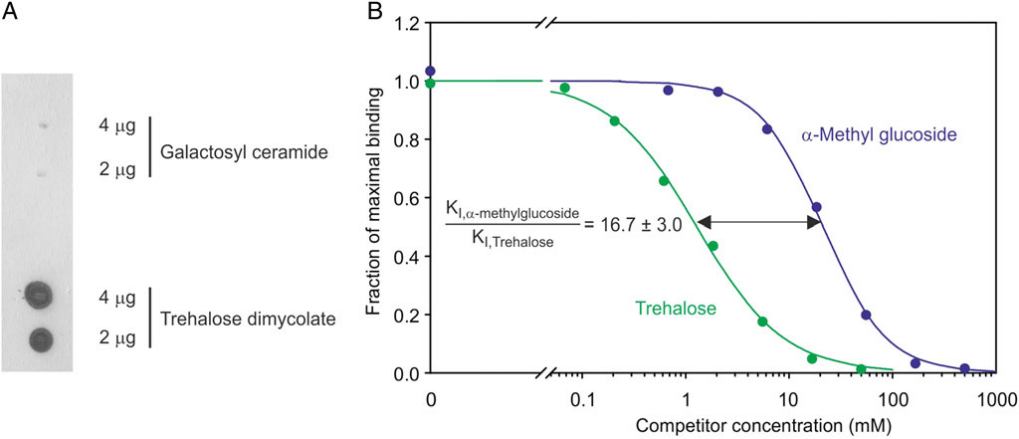
Binding of trehalose-containing ligands to the CRD from human mincle. (**A**) Detection of trehalose dimycolate binding by glycolipid blotting. Glycolipids spotted on PVDF membrane were probed with biotin-tagged mincle CRD followed by alkaline phosphatase-conjugated avidin. (**B**) Quantification of trehalose binding to immobilized mincle CRD by competition with ^125^I-labelled Man_31_BSA. Experimental data are represented by dots and curves obtained by nonlinear least-squares fitting are shown as solid lines. Mean ± SD for the ratio of the *K*_I_ values derived from four replicate experiments is indicated.

The ability of the expressed CRD from human mincle to bind to trehalose dimycolate was verified using a dot blot. The glycolipid was spotted onto PVDF membrane, which was probed sequentially with the biotin-tagged CRD followed by alkaline phosphatase-conjugated avidin (Figure 3A). Specificity was demonstrated by the absence of binding to a control glycolipid. The qualitative preferential binding of the CRD to trehalose demonstrated by the affinity chromatography experiments was also quantified using a binding competition assay. Although mannose is not an optimal ligand for the CRD, it was shown that radioiodinated mannose-conjugated bovine serum albumin (BSA) bearing 30–33 mannose residues per mole of protein binds to the biotin-tagged CRD immobilized on streptavidin-coated plates. Saturation binding was not achieved, but binding at subsaturating levels of reporter ligand is actually preferred in this situation, so that the resulting *K*_I_ values can be used as an approximation for the affinity constants of competing ligands (Levitzki 1997). Binding competition studies revealed a 16.7-fold higher affinity for trehalose compared with α-methylglucoside (Figure 3B).

### Analysis of trehalose binding site in human mincle

**Fig. 4.**
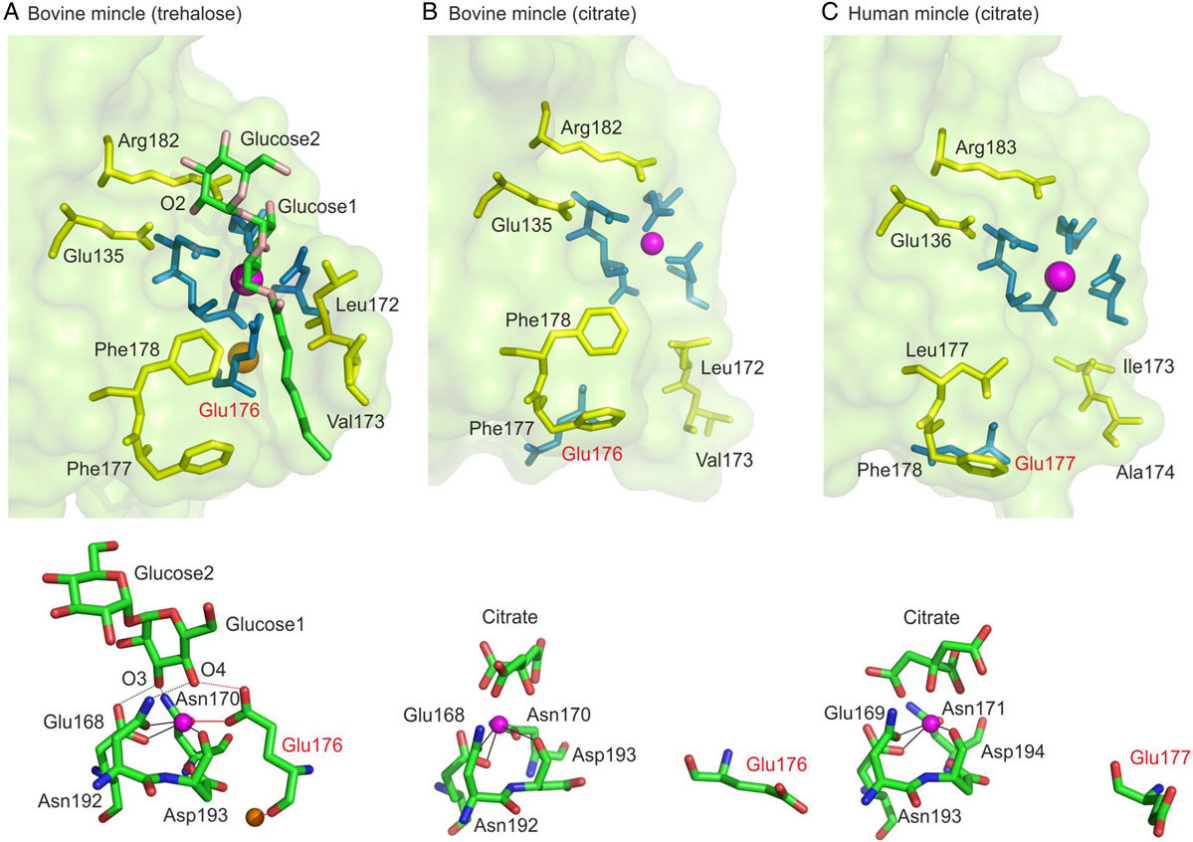
Structures of CRDs from mincle. Upper panels show the overall binding site, whereas lower panels show the residues that ligate to the conserved Ca^2+^. (**A**) Structure of bovine mincle with trehalose bound (PDB 4kzv). Side chains highlighted in green form the primary sugar-binding site by ligating to the conserved Ca^2+^, shown in magenta, and by making hydrogen bonds with 3- and 4-OH groups in the first glucose residue in trehalose. Side chains that form hydrogen bonds with the 2-OH group of the second glucose residue are highlighted in yellow, as are side chains that are proposed to form a binding site for the acyl chain attached to trehalose. A secondary cation near the sugar-binding site is shown in orange. This cation is Na^+^ in the previously reported structure of trehalose bound to bovine mincle (Feinberg et al. 2013), but similar crystals in which Ca^2+^ is present at this site have been analyzed subsequently (Hadar Feinberg, Kurt Drickamer and William I. Weis, unpublished observations). (**B**) Structure of bovine mincle with citrate bound (PDB 4kzw). Residues from the binding site in (A) are shown using the same color scheme. Key differences in the structure result from displacement of loops of protein containing Ca^2+^ ligand Glu135 and residues Leu172 and Val173, which form one side of the hydrophobic groove in the ligand-bound conformation. Citrate is omitted from the top panel for clarity. (**C**) Structure of human mincle (PDB 3WH2). Highlighting of residues is as in (A). The loops of protein containing potential Ca^2+^ ligand Glu136 and residues Ile173 and Ala174, corresponding to residues Glu135, Leu172 and Val173 in bovine mincle, show the conformation very similar to those seen in (B). Citrate is omitted from the top panel for clarity. This figure was created using PyMol.

Key information about the mechanism of trehalose dimycolate binding to bovine mincle was previously obtained from a cocrystal of the CRD with trehalose (Feinberg et al. 2013). This structure revealed that one glucose residue of the trehalose is bound to Ca^2+^ in the conserved site that is present in all sugar-binding C-type CRDs and that additional specificity and affinity results from interaction of the second glucose residue with an adjacent secondary site (Figure 4A). Two structures have been reported for the CRD from human mincle (Furukawa et al. 2013). The two structures differ by the presence or absence of a citrate ion bound to the conserved Ca^2+^, but the protein conformation is the same in the two crystals. Although this conformation is similar to that observed for a bovine mincle complex with trehalose, the human structure differs in some key respects and more closely resembles an alternative conformation of the bovine CRD with a bound citrate ion (Figure 4B and C).

**Fig. 5.**
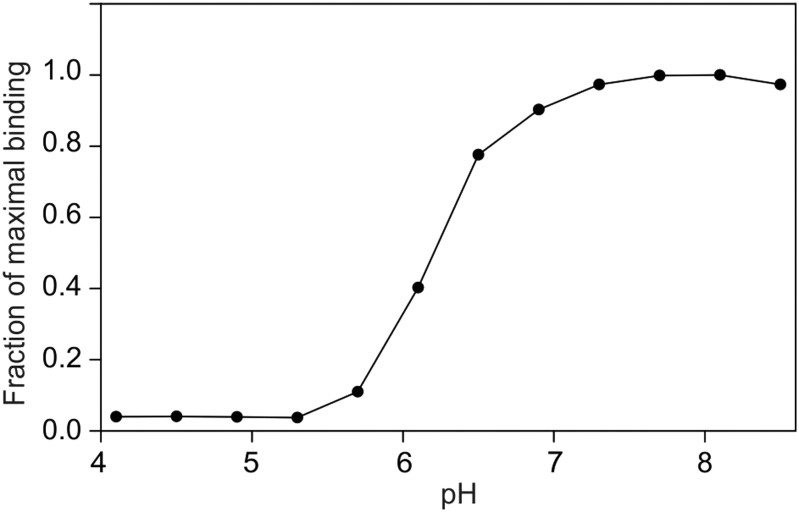
pH dependence of ligand binding to human mincle. Binding of ^125^I-mannose-BSA to the biotin-tagged CRD immobilized on streptavidin-coated wells was measured in buffers containing 150 mM NaCl, 3 mM CaCl_2_, 25 mM 2-(*N*-morpholino)ethanesulfonic acid and 25 mM 3-(*N*-morpholino)propanesulfonic acid at various pH values. Following incubation with ligand for 2 h at 4°C, plates were washed 3× with binding buffer containing 150 mM NaCl, 3 mM CaCl_2_ and 25 mM Tris–Cl, pH 7.8. The bound radioactivity was normalized to the maximal binding observed at pH 7.7–8.1.

In the primary sugar-binding site in bovine mincle, five amino acid side chains ligate the conserved Ca^2+^, forming a series of coordination and hydrogen bonds that are the same as those seen in numerous other C-type CRDs, including those from mannose-binding protein (Weis et al. 1992), DC-SIGN (Feinberg et al. 2001), the asialoglycoprotein receptor (Meier et al. 2000) and the scavenger receptor C-type lectin (Feinberg et al. 2007). In the human mincle structure, which is based on crystals grown at pH 4.0, the position of four of these residues is conserved, but residue Glu177 is displaced away from the Ca^2+^ (Figure 4C) (Furukawa et al. 2013). A very similar arrangement was observed in the crystals of bovine mincle CRD grown at pH 5.0 in the absence of trehalose (Figure 4B) (Feinberg et al. 2013). The rearrangement of the corresponding residue Glu176 is correlated with the absence of an accessory cation, which is ligated to the main chain carbonyl group of this residue in the binding conformations of the C-type CRDs (Weis et al. 1992; Meier et al. 2000; Feinberg et al. 2001; Feinberg et al. 2007). Loss of a cation at the accessory position in bovine mincle is probably triggered by a decrease in pH as reflected in the loss of sugar-binding activity at pH <6 (Feinberg et al. 2013). pH-dependent loss of Ca^2+^-binding activity has been described as part of the pH-sensitive switch that allows endosomal release of ligands from C-type CRDs (Feinberg et al. 2000; Feinberg et al. 2007). A nearly identical pH profile for ligand-binding activity is seen for human mincle (Figure 5), suggesting similar dependence on the presence of bound cations.

**Fig. 6.**
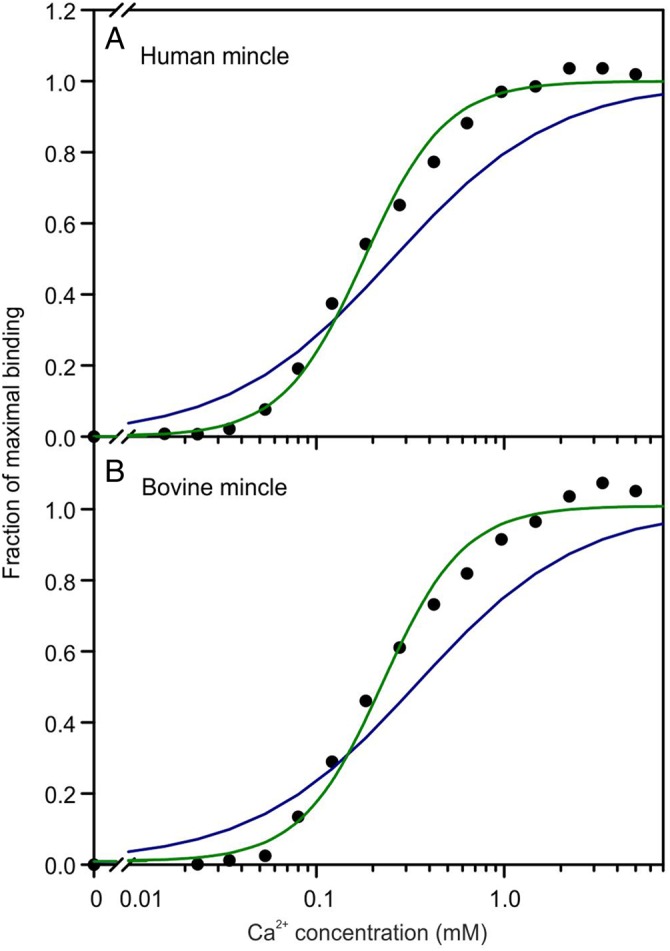
Ca^2+^ dependence of ligand binding to human and bovine mincle. Binding of ^125^I-mannose-BSA to the biotin-tagged CRDs immobilized in streptavidin-coated wells was quantified for (**A**) the CRD from human mincle and (**B**) the CRD from bovine mincle. Binding was conducted for 2 h in the presence of 150 mM NaCl and 25 mM Tris–Cl, pH 7.8 at 4°C. Following incubation with ligand, wells were washed 3× with buffer containing 150 mM NaCl, 25 mM Tris–Cl, pH 7.8, and 25 mM CaCl_2_. The experimental data, shown as black spheres, were fitted to first- and second-order binding equations, shown respectively as blue and green lines, using a nonlinear least-squares fitting program.

The role of multiple Ca^2+^ in the binding site of the human and bovine mincle CRDs was compared directly by conducting binding assays in the presence of different Ca^2+^ concentrations (Figure 6). The results for the two proteins are nearly identical. In both cases, the Ca^2+^ dependence of mannose-BSA binding is best fitted with a second order equation. Based on the structure of the ligand-bound form of bovine mincle, the fact that the Ca^2+^ dependence is higher than first order can be explained by the presence of the accessory Ca^2+^ required for the correct positioning of the bridging glutamic acid residue at position 176. The very close similarity in the Ca^2+^ dependence of human mincle strongly suggests that the ligand-binding conformation of this CRD would also involve a second Ca^2+^ in the binding site. Binding of trehalose to the bovine CRD is able to shift the position of Glu135 in order to achieve a ligand-binding conformation, but the fact that the human mincle crystals were grown at pH 4.0 probably makes this transition energetically unfavorable for these crystals.

In addition to coordination and hydrogen bonds involving the 3- and 4-hydroxyl groups of glucose in the primary binding site of bovine mincle, there are potential packing interactions of the side chain of Leu172 with the A face of the glucose residue. In human mincle, the corresponding residue is Ile173. An isoleucine side chain would be able to make similar packing interactions, but in the human mincle structures this residue is not positioned adjacent to the primary binding site because of the absence of the accessory Ca^2+^. The fact that the affinity of human mincle CRD for glucose is similar to that seen for the bovine CRD (Figure 3B) is consistent with the suggestion that, in the binding conformation, both Glu136 and Ile173 would be positioned to interact with the sugar in the primary binding site. Taken together, these results suggest that the crystals of the human mincle CRD represent a low pH form that differs from the ligand-binding conformation.

**Fig. 7.**
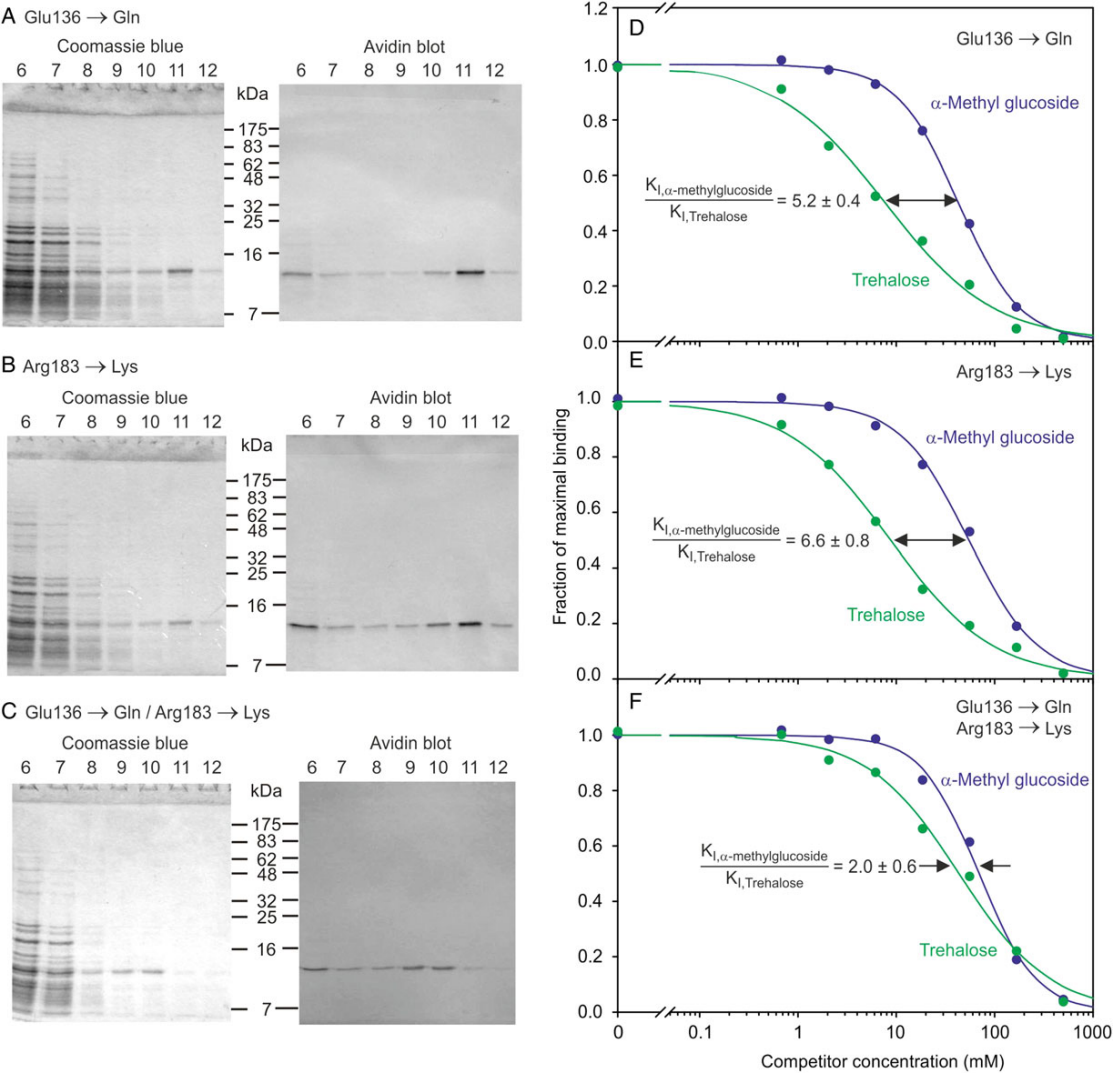
Mutational analysis of the role of the secondary sugar-binding site that enhances trehalose binding to human mincle. (**A**–**C**) Purification of mutant proteins on invertase-Sepharose. Fractions eluted from the column were analyzed on parallel SDS–polyacrylamide gels that were stained with Coomassie blue or blotted and probed with alkaline phosphatase-conjugated avidin. (**D**–**F**) Comparison of binding of mutant proteins to trehalose and α-methyl glucoside using binding competition assay. Experimental data are represented by dots and curves obtained by nonlinear least-squares fitting are shown as solid lines. Mean ± SD for the ratios of the *K*_I_ values derived from three replicate experiments are indicated.

In spite of the differences in the conformation of the primary sugar-binding site observed in the human mincle structure, it appears that the secondary sugar-binding site is preserved. Residues Glu136 and Arg183 in the human protein are in nearly identical positions to the corresponding residues Glu135 and Arg182 in the bovine protein. These residues form hydrogen bonds with the 2-OH group of the second glucose residue (Figure 4A). In order to investigate the way that trehalose binds to human mincle, these two residues were mutated to glutamine and lysine, respectively, either a single or a double mutation. A decrease in the affinity for trehalose of mutant CRDs with these changes was evident from the observation that they do not bind efficiently to the trehalose-Sepharose resin (data not shown). For these proteins, purification was achieved by using a column of immobilized yeast invertase, which has previously been used to purify proteins with weak affinity for sugars (Taylor and Drickamer 2003) and by limiting the extent of washing of the column before elution with EDTA (Figure 7A–C). The ability of the purified mutant proteins to bind trehalose was assessed quantitatively using the binding competition assay (Figure 7D–F). The individual mutations result in decreased affinity for trehalose compared with the affinity for α-methyl glucoside, which can only bind at the primary binding site, and the double mutant is reduced to an affinity only twice that of methyl glucoside, corresponding to the fact that there are two glucose residues in the trehalose molecule. The results demonstrate that these two residues constitute a secondary binding site that interacts with the second glucose residue in trehalose. Thus, the role for the secondary binding site in human mincle is analogous to that seen for bovine mincle and these results provide further evidence that the mechanism of binding is very similar in the two proteins.

### Binding of acylated trehalose structures

**Fig. 8.**
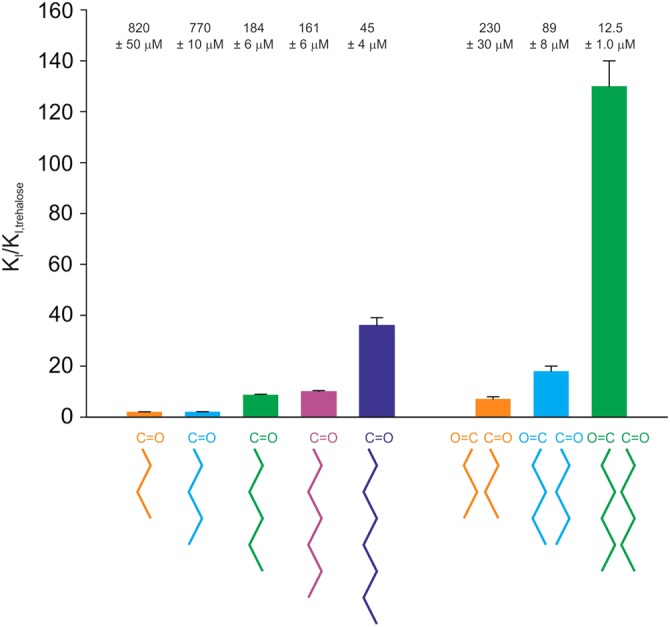
Binding of mono- and diacyl derivatives of trehalose to human mincle CRD. Binding competition studies were conducted with the biotin-tagged CRD immobilized on streptavidin-coated plates and ^125^I-mannose-BSA used as the reporter ligand in the presence of increasing concentrations of the competing trehalose derivatives. *K*_I_ values were determined by nonlinear least-squares fitting. *K*_I_ values relative to the *K*_I_ for trehalose are plotted, with the absolute affinities at the top and structures of acyl groups attached to the 6-OH groups shown below. Mean values for the ratios of the *K*_I_ values derived from 3 to 4 replicate experiments are shown as bars, with the SD for each indicated by the error bars.

A further feature of the ligand-binding site in bovine mincle is the adjacent hydrophobic region that has been postulated to be involved in binding of the mycolic acid groups that are attached to the 6-OH groups of trehalose. Increased affinity of human mincle for trehalose with extended acyl groups on the 6-OH groups was demonstrated using a series of mono- and diacylated derivatives of trehalose prepared from free sugar and acids using a lipase operating under anhydrous conditions (Figure 8). As in the case of the bovine protein, affinity increases for human mincle were observed as the length of a single attached acyl chain was increased from 3 to 8 carbons. The increases in affinity compared with trehalose are quantitatively very similar for bovine and human mincle, consistent with the suggestion that the binding mechanism is similar. Three derivatives with acyl groups attached to both sugar residues were synthesized and tested for binding in the competition assay. Each of the diacyl compounds binds with substantially higher affinity than monoacyl compounds with the same length of acids attached. The absolute affinity for the dihexanoate derivative is 12.5 μM, making it an unusually high-affinity low-molecular weight, monovalent ligand for a C-type CRD.

**Fig. 9.**
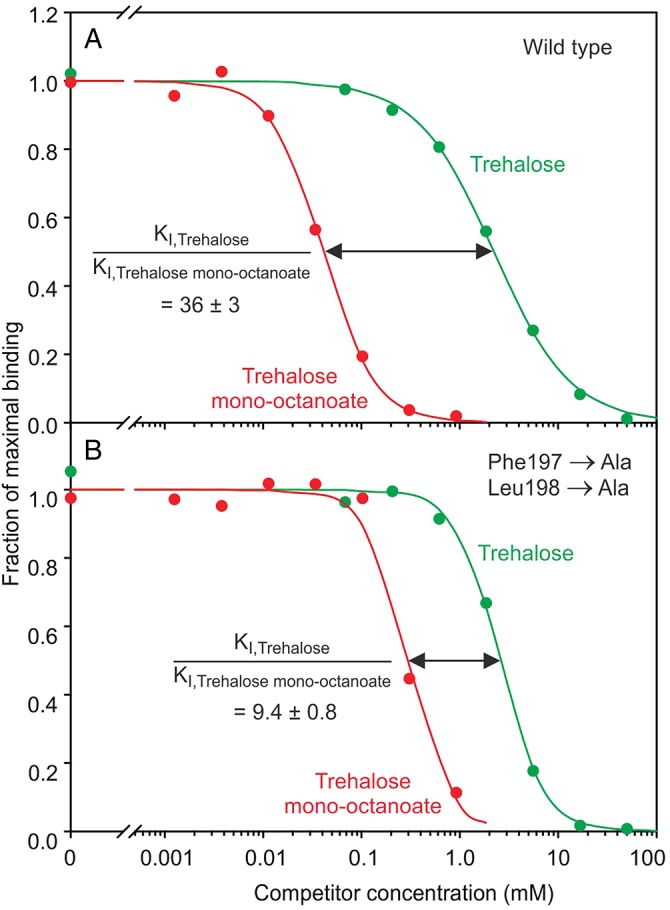
Mutagenesis of residues involved in binding of human mincle to acyl groups attached to trehalose. (**A**) Wild-type CRD probed. (**B**) Mutant in which alanine residues have been introduced at positions corresponding to Phe197 and Leu198. Experimental data are represented by dots and curves obtained by nonlinear least-squares fitting are shown as solid lines. Mean ± SD for the ratios of the *K*_I_ values derived from three replicate experiments are indicated. The double mutation results in loss in affinity for monooctanoyl trehalose compared with free trehalose.

For bovine mincle, it was specifically suggested that a hydrophobic groove, running between residues Phe197 and Phe198 on one side and Leu172 and Val173 on the other, would accommodate a fatty acid chain, leading to increased affinity for acylated derivatives of trehalose (Figure 4A). In the crystal structures of the CRD from human mincle, residues Leu173 and Phe174 occupy positions very similar to the two phenylalanine residues on one side of the groove in the bovine protein, suggesting that they could form a groove analogous to that seen in bovine mincle. The importance of these residues in binding of the acyl derivatives of trehalose was tested by mutagenesis of both side chains to alanine. The resulting protein shows a relative loss in affinity for the monohexanoate derivative similar to that which results from removal of the two corresponding residues in bovine mincle (Figure 9).

These findings suggest a similar mechanism for binding acyl chains attached to trehalose in bovine and human mincle. However, in contrast to residues on the left of the groove in Figure 4, residues Ile173 and Ala179 in human mincle are shifted away from the binding site compared with the corresponding residues in bovine mincle, which form the right side of the groove. Based on the considerations presented above, it can be suggested that rearrangements would occur on binding of an accessory Ca^2+^, leading to formation of a groove structure. However, because of the potential role of Ile173 in the primary binding site and the fact that residue 179 is already an alanine residue, it was not readily possible to test this suggestion by further mutagenesis.

## Discussion

The biochemical studies reported here demonstrate very close similarities between the binding properties of the CRDs from human and bovine mincle. Similarities include nearly identical pH and Ca^2+^ dependence of ligand binding as well as very similar affinity enhancements for trehalose compared with glucose and for acylated versions of trehalose compared with trehalose. These results indicate that the primary binding site for one glucose residue, as well as the accessory binding sites for the second glucose residue and the acyl chain attached to the sugar are very similar. The effects of mutations in these secondary binding sites provide further evidence for analogous interactions with ligands.

In spite of these similarities, the recently reported structure of the human mincle CRD appears to differ significantly from the structure of the bovine CRD (Furukawa et al. 2013). However, a second structure for the bovine CRD suggests that there is an alternative conformation of the CRD, which is very similar to the structure reported from human mincle. The key difference between these conformations is the absence of a secondary cation in the unliganded structure, which results in a major shift in the position of residue Glu135 in the bovine protein. In the ligand-bound form of bovine mincle, as well as in many other C-type CRDs with bound sugar ligands (Weis and Drickamer 1996; Feinberg et al. 2013), this glutamic acid residue bridges between this secondary cation and the Ca^2+^ in the primary binding site, since the main chain carbonyl group is a ligand for the secondary cation and the side chain carboxyl group is a ligand for the binding site Ca^2+^ and also makes hydrogen bonds with the bound sugar residue.

It is important to note that the structures of both human and bovine mincle CRDs in which the glutamic acid residue is displaced away from the primary binding site were obtained at low pH. Many receptors that contain C-type CRDs are recycling endocytic receptors that must release their ligands at endosomal pH, so a conformational change under such circumstances would be expected. Such changes, including displacement of the bridging glutamic acid residue, have been observed for such endocytic receptors (Feinberg et al. 2000; Feinberg et al. 2007). In common with these receptors, both bovine and human mincle lose binding activity at low pH. For bovine mincle, the structure with bound sugar ligand was obtained in the presence of high concentrations of trehalose. Forcing the transition into the sugar-binding conformation would likely have been difficult for the human crystals, since these were obtained at significantly lower pH (Furukawa et al. 2013).

Taken together, the results presented here suggest that the reported structure of human mincle is likely to represent an inactive conformation rather than a sugar-binding conformation observed at the cell surface. Based on the binding and mutagenesis studies, the sugar-binding conformation is likely to resemble closely that of the bovine CRD with bound trehalose. These findings have important implications for exploiting the properties of mincle in developing potential ways of blocking the interaction with mycobacterial ligands during human infections with *Mycobacterium tuberculosis* as well as agriculturally important infections of cows with *M. bovis*, since approaches that are developed for either protein will also be applicable to the other.

## Materials and methods

### Cloning and mutagenesis

A cDNA encoding the CRD from human mincle was amplified from a placental cDNA library (Clontech) using primers to introduce restriction sites at the ends. PCR amplification was performed with Advantage 2 polymerase mix (Takara) and the resulting DNA fragments were isolated by agarose gel electrophoresis and cloned into the pCR2.1-TOPO vector (Invitrogen). The sequence was confirmed on an Applied Biosystems 310 genetic analyzer. The inserts were excised and inserted into the pompA-derived and pT5T expression vectors (Eisenberg et al. 1990; Taylor and Drickamer 2003). For expression of biotin-tagged proteins, nucleotides encoding the biotinylation sequence were appended at the 3′ end of the coding sequence by reamplification with appropriate PCR primers. Mutagenesis of codons for individual amino acids was performed by two-step PCR (Cormack 1997) using the wild-type expression clone as template.

### Protein expression and purification

For production of protein by secretion into bacterial periplasm, *Escherichia coli* strain JA221 containing the ompA expression vector was grown with shaking at 25°C to an *A*_550_ of 0.8. Protein expression was induced with 50 μM isopropyl-β-d-thiogalactoside and CaCl_2_ was added to a final concentration of 100 mM. After growth at 25–30°C for a further 18 h, bacteria were harvested by centrifugation at 4000 × *g* for 15 min at 4°C. The pellets were resuspended in 25 mL of cold loading buffer (0.15 M NaCl, 25 mM Tris–HCl, pH 7.8, 25 mM CaCl_2_) and lysed by sonication. Lysed bacteria were centrifuged at 10,000 × *g* for 15 min and the supernatant was recentrifuged at 100,000 × *g* for 30 min at 4°C. The supernatant was applied to 10-mL columns of maltose- or trehalose-Sepharose prepared by the divinyl sulfone method (Fornstedt and Porath 1975). After rinsing with 14 mL of loading buffer, the bound protein was eluted with 16×1 mL eluting buffer (150 mM NaCl, 25 mM Tris–Cl, pH 7.8, 2.5 mM EDTA). Fractions containing the CRD were identified by examining aliquots on 17.5% SDS–polyacrylamide gels that were stained with Coomassie Blue.

Biotin-tagged human mincle CRD was expressed from the pT5T plasmid in *E. coli* strain BL21(DE3) containing the pBirA plasmid, which encodes biotin ligase (Schatz 1993), at 37°C with shaking to an *A*_550_ of 0.7. Isopropyl-β-d-thiogalactoside was added to a concentration of 100 mg/L followed by growth for a further 150 min at 37°C. Bacteria harvested by centrifuging at 4000 × *g* for 15 min at 4°C were washed by resuspending in 10 mM Tris–HCl, pH 7.8, centrifuged at 10,000 × *g* for 10 min at 4°C, resuspended in 30 mL of 10 mM Tris–HCl, pH 7.8 and lysed by sonication. Inclusion bodies were isolated by centrifugation at 10,000 × *g* for 15 min at 4°C. Inclusion bodies from 6 L of bacterial culture were dissolved in 100 mL of 6 M guanidine HCl containing 100 mM Tris–Cl, pH 7.8, and incubated in the presence of 0.01% 2-mercaptoethanol for 30 min at 4°C. Following centrifugation for 30 min at 100,000 × *g*, the supernatant was dialyzed against three changes of 0.5 M NaCl, 25 mM Tris–Cl, pH 7.8, 2.5 mM EDTA at 4°C. Insoluble material was removed by centrifugation. For the wild-type CRD, affinity chromatography was performed as for the periplasmic protein and for mutant proteins in which the secondary binding site is compromised, the column was rinsed with only 2 mL of loading buffer.

### Binding competition assays

Plates of removable polystyrene wells coated with streptavidin (Thermo Scientific Pierce) were washed 3× with binding buffer (150 mM NaCl, 25 mM Tris–Cl, pH 7.8, 2.5 mM CaCl_2_), incubated for 2 h at 4°C with biotin-tagged CRDs at 5 μg/mL in 0.1% (w/v) BSA in binding buffer and again washed 3× with binding buffer. Serial 3-fold dilutions of ligands were made and buffer and reporter ligand were added so that the final concentrations corresponded to 0.5 μg/mL ^125^I-Man_31_-BSA in 0.1% BSA in binding buffer. After incubation for 2 h at 4°C, wells were washed 3× with binding buffer and counted in a Wallac WIZARD gamma counter (PerkinElmer Life Sciences). Inhibition constants were obtained by fitting to a simple binding curve (Stambach and Taylor 2003) using SigmaPlot. Ratios of different competing ligands were calculated for assays performed together on one assay plate. Values reported are means ± SD for 3–4 replicate experiments.

### Ca^2+^ and pH dependence of ligand binding

Ca^2+^ dependence of ligand binding was measured in the same assay format as for the competition assays but with no competing ligand and with buffers containing 0.15 M NaCl, 25 mM Tris–Cl, pH 7.8, and CaCl_2_ concentrations ranging from 0 to 5 mM generated by serial 1.5-fold dilutions. For pH dependence assays, a series of buffers containing 25 mM 2-(*N*-morpholino)ethanesulfonic acid and 25 mM 3-(*N*-morpholino)propanesulfonic acid were used in place of the Tris buffer.

### Glycolipid blotting

For glycolipid blotting, 1–2 μL of trehalose dimycolate (Carbosynth Ltd.) at 2 μg/μL in chloroform and galactosylceramide from bovine brain (Sigma) at 2 μg/μL in chloroform–methanol (2 : 1) were spotted on polyvinylidine difluoride membrane (Millipore). The membrane was dried, wetted with methanol and blocked in 5% (w/v) BSA in binding buffer for 1 h at room temperature with shaking. The blot was incubated for 1.5 h with 1 μg/mL biotin-tagged CRD from human mincle in 5% (w/v) BSA in binding buffer, washed 3× 5 min with binding buffer and incubated with 0.5 μg/mL alkaline phosphatase-conjugated extravidin (Sigma) in 5% (w/v) BSA in binding buffer for 1 h at room temperature. After 3× 5 min washes with binding buffer, the blot was developed for 2 min with nitro blue tetrazolium and 5-bromo-4-chloro-3-indolyl phosphate substrate (Millipore).

### Synthesis of acylated trehalose derivatives

Trehalose derivatives were synthesized using the lipase from *Candida antarctica* (Sigma Chemical Co.) as previously described (Feinberg et al. 2013). In order to achieve efficient synthesis of diacylated derivatives of trehalose, 500 mg of trehalose was reacted with 5 mL of carboxylic acid in the presence of 500 mg of lipase immobilized on polystyrene beads in 25 mL of isoamyl alcohol. Characterization of compounds that have not previously been described, by NMR and mass spectrometry, is provided in Supplementary Figures S1 and S2.

## Supplementary data

Supplementary data for this article are available online at http://glycob.oxfordjournals.org/.

## Funding

Biotechnology and Biological Sciences Research Council (grant BB/K007718/1 to M.E.T. and K.D.) and the Wellcome Trust (grant 093599 to M.E.T. and K.D.). Funding to pay the Open Access publication charges for this article was provided by the Biotechnology and Biological Sciences Research Council and the Wellcome Trust.

## Conflict of interest

None declared.

## Abbreviations

BSA, bovine serum albumin; CRD, carbohydrate-recognition domain; DC-SIGN, dendritic cell-specific intercellular adhesion molecule-3 grabbing nonintegrin.

## Supplementary Material

Supplementary Data
