# Supplementary material for: Defining the conformation of human mincle that interacts with mycobacterial trehalose dimycolate
Source: Glycobiology. 2014 Jul 15;24(12):1291–300. doi: 10.1093/glycob/cwu072 (PMC4211601; doi:10.1093/glycob/cwu072)
Supplement: Supplementary Data [file supp_24_12_1291__index.html]

Defining the conformation of human mincle that interacts with mycobacterial trehalose dimycolate — Defining the conformation of human mincle that interacts with mycobacterial trehalose dimycolate — Supplementary Data 

# Defining the conformation of human mincle that interacts with mycobacterial trehalose dimycolate

## Supplementary Data

Supplementary Data

**Files in this Data Supplement:**

- Supplementary Data - Pdf file
