# Supplementary material for: Defining the conformation of human mincle that interacts with mycobacterial trehalose dimycolate
Source: Glycobiology. 2014 Jul 15;24(12):1291–300. doi: 10.1093/glycob/cwu072 (PMC4211601; doi:10.1093/glycob/cwu072)

Supplemental Information

Mechanism of interaction of human mincle with mycobacterial trehalose dimycolate probed using synthetic analogues

Sabine A. F. Jégouzo, Edward Harding, Oliver Acton, Maximus J. Rex, Andrew J. Fadden, Maureen E. Taylor, Kurt Drickamer

Figure S1. Mass spectrometry of novel di-acylated trehalose derivatives.

Figure S2. Proton NMR spectra of novel di-acylated trehalose derivatives.

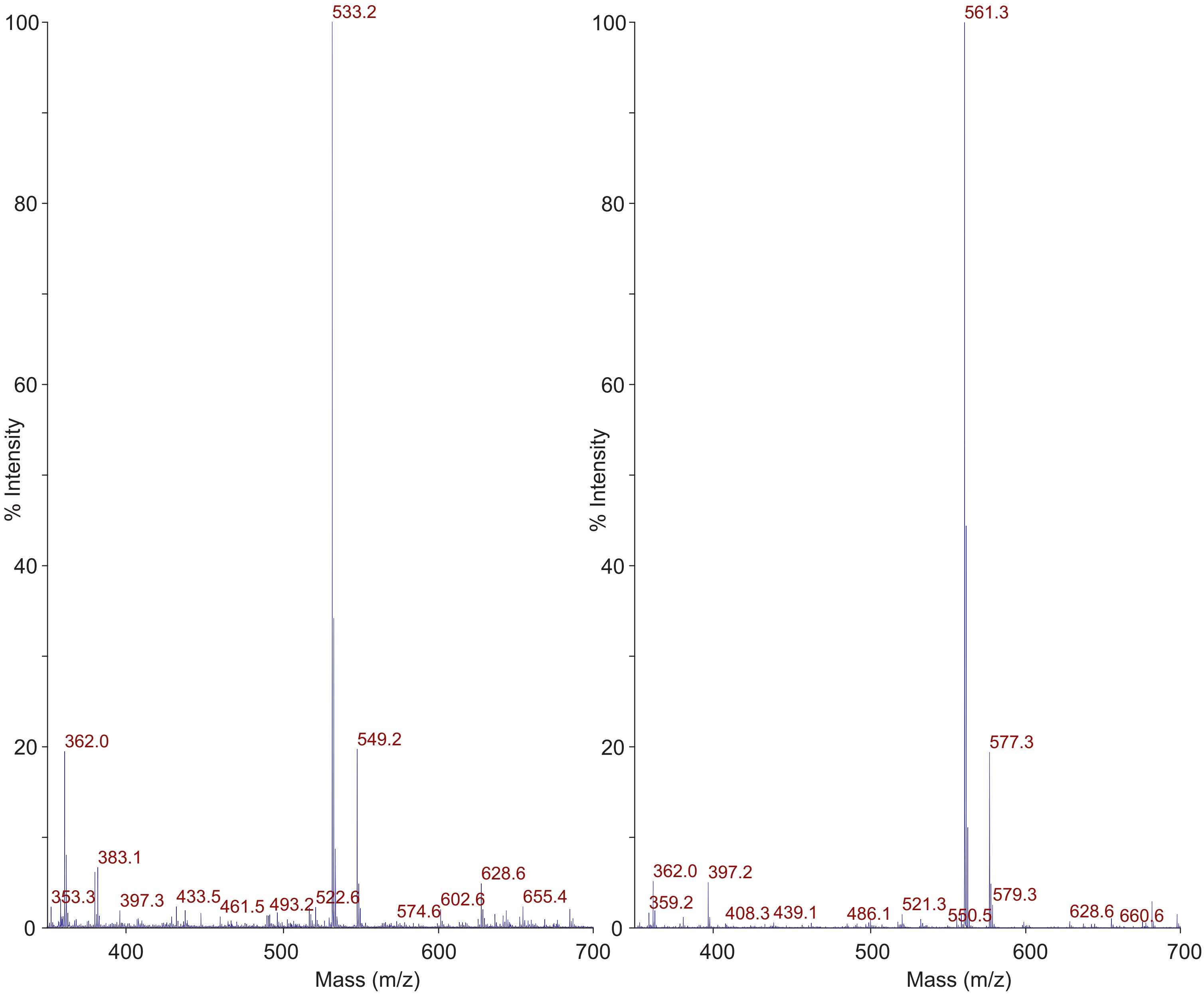

A Trehalose divalerate 533 (Na) / 549 (K)
[Monovalerate 449 (Na)]

B Trehalose dihexanoate 561 (Na) / 577 (K)
[Monohexanoate 463 (Na)]

Figure S1. Mass spectrometry of di-acylated trehalose derivatives. Samples were prepared by spotting together equal volumes of matrix, 2,5-dihydroxybenzoic acid as a 10 mg/ml solution 80% methanol, and sugar derivative, dissolved at approximately 1 mg/ml in water, on a target plate. Mass spectrometry was performed on an Applied Biosystems 4800 matrix assisted-laser desorption time-of-flight mass spectrometer.

Figure S2. Proton NMR spectra of diacylated trehalose derivatives. Samples for NMR spectra, dissolved in D_2O at approximately 5 mg/ml, were analyzed on a Bruker 400 MHz spectrometer. Spectra are annotated to show signals from the acyl side chain (letters A-G) and key signals from trehalose.

A Trehalose divalerate

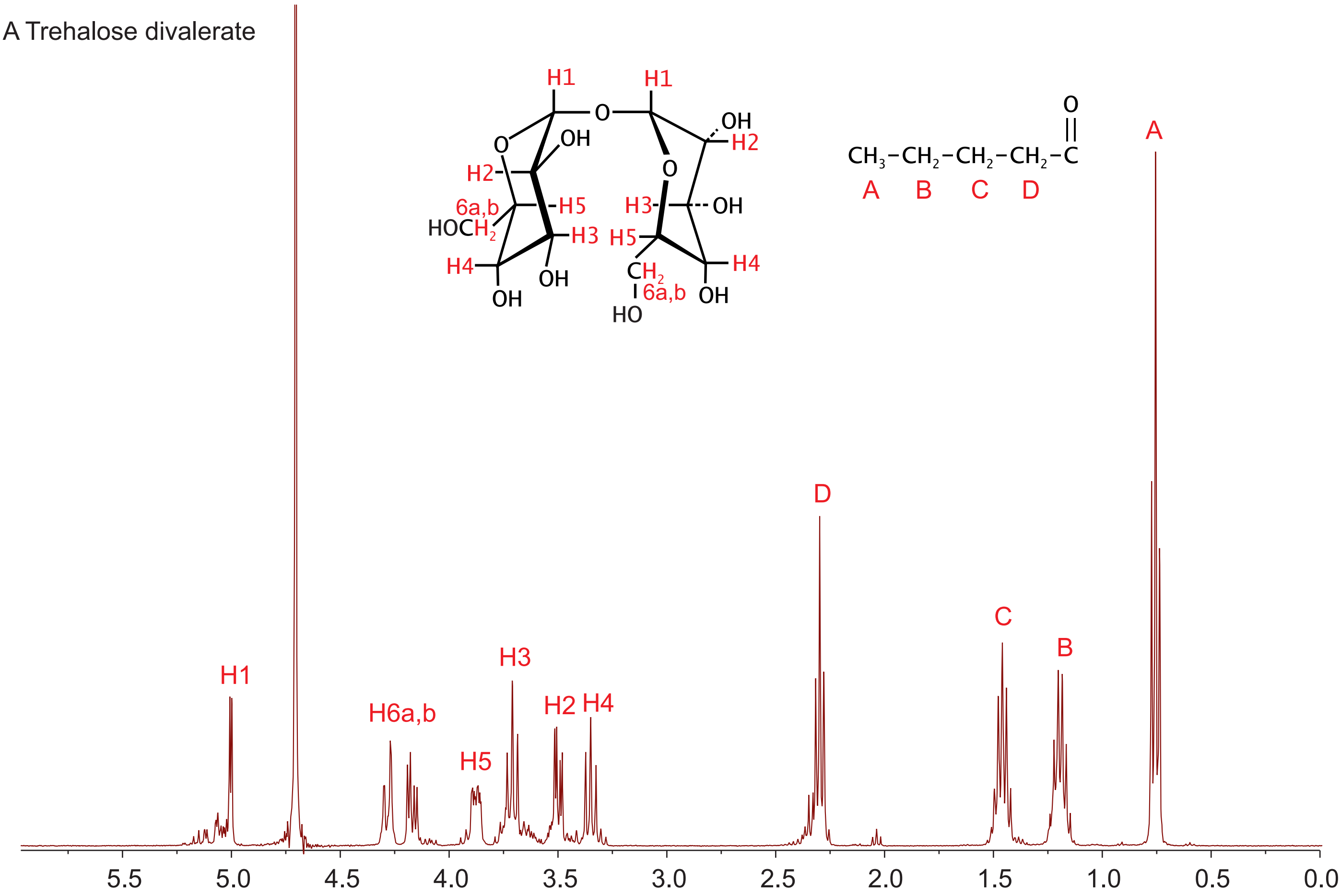

B Trehalose dihexanoate

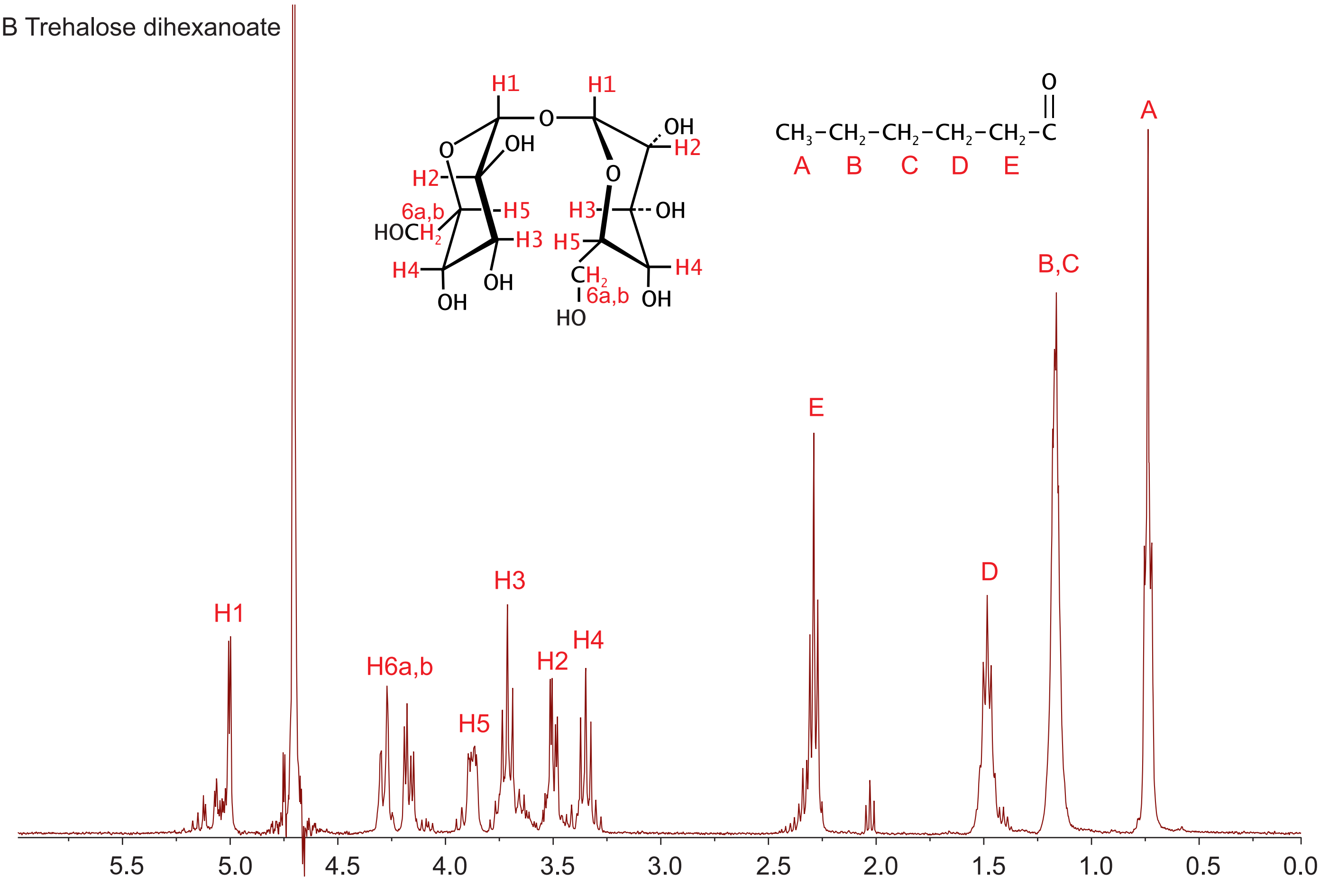

Supplement: Supplementary Data [file supp_cwu072_cwu072supp.pdf]
